# "I know I have arthritis but I don't know what type". Understanding and knowledge of this chronic condition

**DOI:** 10.1186/1471-2474-11-174

**Published:** 2010-08-06

**Authors:** Tiffany K Gill, Catherine L Hill, Robert J Adams, Danny Broderick, Julie Black, Anne W Taylor

**Affiliations:** 1 Population Research & Outcomes Studies Unit, SA Health, Adelaide, SA, Australia; 2 Department of Medicine, University of Adelaide, Adelaide, SA, Australia; 3 Rheumatology Unit, The Queen Elizabeth Hospital, Woodville, SA, Australia; 4 The Health Observatory, The University of Adelaide, The Queen Elizabeth Hospital Campus, Woodville, SA, Australia; 5 Public Health and Clinical Coordination, SA Health, Adelaide, SA, Australia; 6 Arthritis Foundation of South Australia, Adelaide, SA, Australia

## Abstract

**Background:**

"Arthritis" is a common musculoskeletal condition but the knowledge of what type of arthritis people have, may be limited but may have changed over time in response to campaigns, increased awareness and improved health literacy. This paper describes people who did not know what type of arthritis they had, by a range of relevant demographic and socioeconomic variables, and assesses changes over time in the proportion of people who report having arthritis but do not know what type, using representative population surveillance data.

**Methods:**

Data were collected using the South Australian Monitoring and Surveillance System (SAMSS), a risk factor surveillance system where each month, a representative random sample of South Australians is selected from the Electronic White Pages, with interviews conducted using computer assisted telephone interviewing (CATI). Data were used for the period January 2006 to December 2008 (n = 16465) for respondents aged 18 years and over.

**Results:**

Overall, the proportion of respondents who did not know what type of arthritis they had, among people aged 18 years and over, for 2006 to 2008 was 6.5% (95% CI 6.1-6.9). When considering only those respondents reporting that they had been told by a doctor that they had arthritis, 30.1% did not know what type of arthritis they had. Multivariate analysis indicated that males, those with have a trade, certificate or diploma or secondary level of education, who spoke a language other than English at home, were widowed and earned $20,001 to $60,000, more than $80,000 or did not state their income were more likely to maintain that they did not know what type of arthritis they had.

**Conclusions:**

Population ageing and an increase in arthritis prevalence in the future will further increase the burden of arthritis. These increases in prevalence are not inevitable, especially if investments are made in public health prevention programs, particularly those addressing cultural and linguistic diversity and differences in socio-economic status and health literacy.

## Background

Chronic disease is a crucial public health issue [1]. Being able to provide policy and planning experts with quality data to assist them in their decision making is important, in an endeavour to provide appropriate preventive and management policies, programs and interventions. In our ageing society, where scarce health resources are being spread increasingly thin, evidence-based, valid, population-wide estimates of chronic conditions are required by decision-makers. The self-report nature of many of the data elements collected using population surveys rely on people understanding their condition. One area of concern is when patients do not understand what has been told to them - even, in some instances, the most basic labelling of the condition they have been diagnosed with. Arthritis is one example of a chronic condition where self-report estimates may be compromised because of the lack of comprehension or understanding associated with the diagnosis.

Health literacy is described as having the ability to perform basic reading and numerical tasks required to function in a health care environment [2]. Health literacy is vital to all people, but people with chronic diseases in particular, to ensure reasonable measures are taken for treatment and maintenance of the condition. Having poor or inadequate health literacy can contribute to a variety of outcomes including: poor compliance, uncontrolled chronic disease, difficulties with accessing health care, following instructions from a physician or taking medication properly, and inability to complete forms [2]. All of these activities are compromised when people do not even know exactly what condition they are dealing with.

Not only has it been shown that there is a significant association between less knowledge of one's illness and lower functional health literacy levels (FHL) [3], it has been documented that patients with inadequate health literacy also have difficulty controlling chronic illnesses [3-5]. Health promotion programs and disease prevention initiatives are less likely to reach those with poor health literacy [5] and lower literacy skills correlate with poorer self-reported health status [6]. Age, education and income level are associated with low health literacy [7] and reading ability is also an indicator of functional literacy skills [8]. In assessing health literacy, it has been shown that the highest level of education completed is often higher than the actual level of literacy [9].

People with less health literacy are less likely to ask questions of clinicians. One of the assumptions of the chronic care model is that the reorganization of health care will lead to more productive interactions with informed, involved patients, leading to better outcomes. Yet policies promoting more choice may run the risk of creating a two-tiered system in terms of access, where health literate individuals are able to exercise greater choice, whilst vulnerable groups, such as the elderly, disabled, less educated, or socially excluded, 'fall through the net'. A lack of clarity around a person's diagnosis and condition makes self-management very problematic.

Arthritis is recognised as a major burden on public health across the western world [10], although diseases with more acute mortality often receive more attention [11]. Arthritis is a highly prevalent condition, particularly for women and those in older age groups [10-14]. As of 2008, it is estimated that arthritis affected approximately three million Australians, 16.5% of the total population [15]. This number has been estimated to be higher in South Australia (SA), with approximately a fifth of the SA population reporting that they have arthritis [16]. In recognition of the health and economic burden arthritic conditions cause, and because of the potential for health gain through prevention and lessening of the impact of the diseases, the Australian Federal Government established arthritis and musculoskeletal conditions as a National Health Priority Area in 2002 [15].

Surveillance data describing chronic conditions is critical for increased recognition of the public health burden of these conditions, formulating health care policy, identifying high-risk groups, developing strategies to reduce the burden, and evaluating progress in disease prevention and control [17]. Effective surveillance of chronic disease prevalence and projections of the number of people expected to have chronic conditions in the future provides important information for the allocation of resources to prevention and health service planning. Surveillance systems such as the SA Monitoring and Surveillance System (SAMSS) [18,19] which has been in place since July 2002, have been instrumental in the surveillance of chronic diseases and associated risk factors in SA. Monitoring chronic conditions is important in SA as this state has an ageing population, with the highest proportion of people aged 85 years and over and the lowest proportion aged 0 to 14 years in Australia in 2007, and a total fertility rate of 1.79 in 2006 [20]. Overall, approximately 73% of the SA population live in the capital city, Adelaide [20], and approximately 26% were born overseas [21].

The aims of this paper are to describe the proportion of people in SA, aged 18 years and over, who acknowledge that they have arthritis, but do not know what type, and to describe the demographic and socioeconomic characteristics of these respondents so that appropriate targeting strategies can be put in place. In addition, changes over time in the proportion of people who report having arthritis but do not know what type, will be assessed.

## Methods

SAMSS is a health surveillance system of randomly selected participants that has been conducted monthly since July 2002 and monitors key indicators for national and state priority health and related issues among South Australians of all ages. SAMSS ensures that appropriate, timely and valid population health information is available to monitor health status, respond to changing population health needs, and support planning, implementation, and evaluation of health services and programs. All households in SA, with a number listed in the Electronic White Pages (EWP) were eligible for selection in the sample. A letter introducing SAMSS and informing people of the purpose of the survey is sent to the household of each selected telephone number. Within each household, the person who had their birthday last is selected for interview (surrogate interviews conducted for those 15 years or under). Approximately 600 interviews per month are conducted in English. Overall, the response rate for SAMSS from June 2002 until December 2008 has been approximately 60-70% each month.

The Computer Assisted Telephone Interview (CATI) system is used to conduct the interviews. At least ten call backs are made to the telephone number selected to interview household members. Replacement interviews for persons who could not be contacted or interviewed are not permitted. Weighting is used to correct for disproportionality of the sample with respect to the population of interest. The data are weighted by age, sex and area of residence to the latest Census or Estimated Residential Population (ERP), to reflect the structure of the population in SA. Probability of selection in the household is calculated based on the number of people in the household and the number of telephone listings in the White Pages.

Respondents to each survey are asked a range of health-related questions. Arthritis prevalence was determined by asking the following question and multiple responses were possible:

Have you ever been told by a doctor that you have arthritis? (If yes, what type?)

• *Yes, Osteoarthritis*

• *Yes, Rheumatoid Arthritis*

• *Yes, Juvenile Rheumatoid Arthritis (JRA)*

• *Yes, other (specify)*

• *No, don't have arthritis*

• *Yes, don't know type*

Socioeconomic status is assessed using the Socioeconomic Index for Areas (SEIFA) Index of Relative Social Disadvantage (IRSD). These values are produced by the Australian Bureau of Statistics to measure socioeconomic status by postcode. IRSD scores have been grouped into quintiles (highest, high, middle, low and lowest) for analysis, where the highest quintile represents postcodes with the highest IRSD scores (most advantaged areas) and the lowest quintile represents postcodes with the lowest IRSD scores (most disadvantaged areas) [22]. Other demographic characteristics of respondents such as age, sex, country of birth, income level and education are also determined and since 2007 respondents have been asked their age when arthritis was diagnosed. Data presented in this paper were analysed using Chi-square tests, t-tests, univariate and multivariate logistic regression using SPSS for Windows Version 15.0 [23]. Variables that were significant at p < 0.25 were included in the initial multivariate analysis [24] with the final model comprising variables that were significant at p < 0.05.

## Results

Overall, of all respondents aged 18 years and over in SA between January 2006 to December 2008, 21.6% (95% CI 20.9-22.2) stated that they had one or more forms of doctor diagnosed arthritis. Multiple responses were possible. The proportion of respondents self-reporting that they had osteoarthritis (OA) was 11.7% (95% CI 11.2-12.2), rheumatoid arthritis (RA) 2.8% (95% CI 2.6-3.1), JRA 0.1% (95% CI 0.06-0.2) and other types of arthritis 0.9% (95% CI 0.8-1.1). Overall, the proportion stating that they did not know what type of arthritis they had, was 6.5% (95% CI 6.1-6.9). This equates to approximately 80,000 South Australians [21]. When considering only the respondents reporting that they had been told that they had arthritis, 30.1% (95% CI 28.6-31.6) did not know what type of arthritis they had. For those with arthritis, the mean age of those reporting that they did not know the type of arthritis was 61.47 years (SD 15.58) and for those who knew their type of arthritis was 62.03 (SD 14.73). There was no significant difference between the groups (t = -0.99, p = 0.32). Information on age when first diagnosed has also been collected since 2007. The mean age of first diagnosis for those who did not know the type of arthritis they had was 49.69 (SD 16.92) and for those who knew the type of arthritis they had, 49.21 (SD 15.98). Again there was no significant difference between the two groups (t = 0.63, p = 0.53)

**Figure 1:**
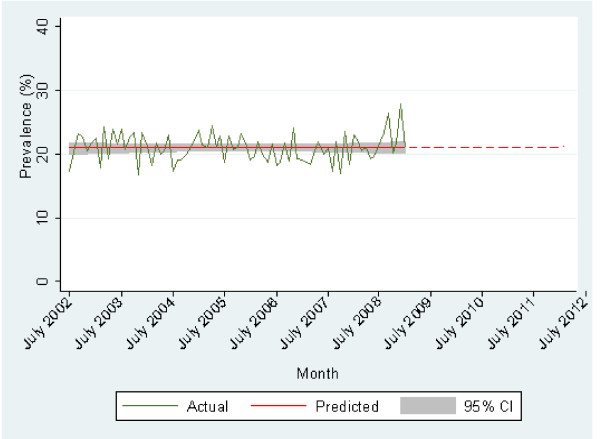
**Prevalence of arthritis over time projected to 2012**.

**Figure 2:**
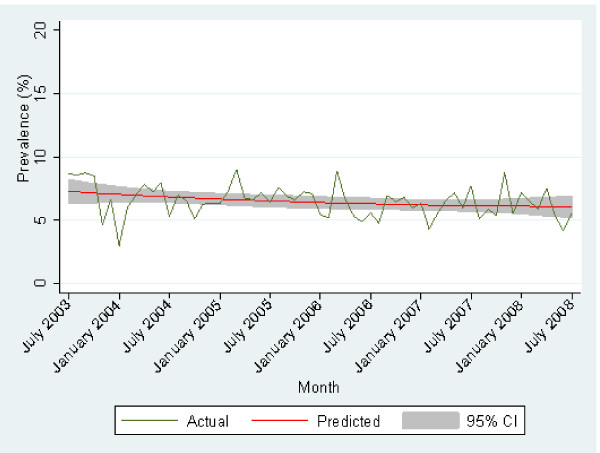
**Prevalence of respondents reporting that they did not know the type of arthritis they had over time**.

The overall trend in the prevalence arthritis since July 2002 is shown in Figure 1. Generally the prevalence of arthritis has remained relatively constant over time. The proportion of respondents reporting an unknown type of arthritis is shown in Figure 2. There has been a slight decline over time.

The characteristics of those unable to name the type of arthritis they had, those who knew the type of arthritis they had and also those without arthritis are presented in Table 1. Those who did not know the type of arthritis were statistically significantly more likely to: be born in a country other than Australia, speak a language other than English, be separated/divorced or widowed, have a gross household income of $40,000 or less per year, be economically inactive (home duties, retired, student, unable to work), be in the lowest two IRSD quintiles or have secondary school as their highest level of educational attainment. In addition, the proportion of respondents who did not know the type of arthritis they have was statistically significantly higher in those aged 60 years or over (χ^2 ^test, p < 0.05) (Table 1).

**Table 1:** Univariate analysis of demographic variables for people with and without arthritis, aged 18 years and over in South Australia, 2006-8

	Arthritis - Don't know type			Arthritis - know type				No arthritis			
	**n**	**%**	**95% CI**	**n**	**%**		**95% CI**	**n**	**%**		**95% CI**

**Sex**											
Male	501	6.2	(5.7-6.8)	909	11.3↓		(10.6-12.0)	6651	82.5↑		(81.7-83.3)
Female	566	6.7	(6.2-7.3)	1574	18.7↑		(17.9-19.6)	6264	74.5↓		(73.6-75.5)

**Age**											
18 to 39 years	90	1.5↓	(1.2-1.8)	181	3.0↓		(2.6-3.5)	5746	95.5↑		(94.9-96.0)
40 to 59 years	371	6.1	(5.5-6.7)	871	14.3↓		(13.5-15.2)	4838	79.6↑		(78.5-80.6)
60 to 79 years	463	13.3↑	(12.2-14.5)	1122	32.2↑		(30.7-33.8)	1897	54.5↓		(52.8-56.1)
80 years and over	144	16.2↑	(14.0-18.8)	309	34.8↑		(31.7-38.0)	435	49.0↓		(45.7-52.3)

Country of birth*											
Australia	783	6.0 ↓	(5.6-6.4)	1828	14.1↓		(13.5-14.7)	10376	79.9↑		(79.2-80.6)
UK/Ireland	138	8.6 ↑	(7.3-10.1)	367	22.8↑		(20.8-24.9)	1103	68.6↓		(66.3-70.8)
Other	142	7.7 ↑	(6.5-9.0)	284	15.4		(13.8-17.1)	1425	77.0		(75.0-78.8)

Language spoken at home*											
English	933	6.3↓	(5.9-6.7)	2227	15.0		(14.4-15.6)	11687	78.7↑		(78.0-79.4)
Other	130	8.1↑	(6.9-9.6)	252	15.7		(14.0-17.6)	1216	76.1↓		(74.0-78.2)

Marital status*											
Married/live with partner	728	6.5	(6.1-7.0)	1731	15.5↑		(14.9-16.2)	8694	78.0↓		(77.2-78.7)
Separated/Divorced	93	8.1↑	(6.7-9.9)	243	21.4↑		(19.1-23.9)	800	70.4↓		(67.7-73.0)
Widowed	172	16.7↑	(14.6-19.1)	369	36.0↑		(33.1-38.9)	486	47.3↓		(44.3-50.4)
Never married	72	2.3↓	(1.8-2.9)	138	4.4↓		(3.7-5.2)	2928	93.3↑		(92.4-94.1)

**Area of residence**											
Metropolitan	758	6.3	(5.9-6.8)	1774	14.8		(14.1-15.4)	9483	78.9↑		(78.2-79.6)
Country	310	7.0	(6.2-7.7)	708	15.9		(14.9-17.0)	3432	77.1↓		(75.9-78.3)

Education*											
Secondary level	690	8.1↑	(7.5-8.7)	1533	18.0↑		(17.2-18.8)	6303	73.9↓		(73.0-74.8)
Trade/Certificate/Diploma	267	6.3	(5.6-7.0)	566	13.3↓		(12.3-14.3)	3429	80.5↑		(79.2-81.6)
Degree or higher	108	3.0↓	(2.5-3.6)	380	10.4↓		(9.5-11.5)	3157	86.6↑		(85.5-87.7)

Work status*											
Full time employed	256	3.6↓	(3.2-4.0)	557	7.8↓		(7.2-8.5)	6320	88.6↑		(87.8-89.3)
Part time	148	4.9↓	(4.2-5.7)	347	11.4↓		(10.3-12.6)	2543	83.7↑		(82.4-85.0)
Unemployed	19	4.7	(3.1-7.3)	34	8.3↓		(6.0-11.4)	354	86.9↑		(83.3-89.9)
Economically inactive	644	10.9↑	(10.2-11.8)	1544	26.2↑		(25.1-27.4)	3698	62.8↓		(61.6-64.1)

**Income**											
Up to $20,000	230	11.9↑	(10.6-13.5)	641	33.3↑		(31.2-35.4)	1053	54.7↓		(52.5-57.0)
$20,001 to $40,000	296	11.1↑	(9.9-12.3)	624	23.3↑		(21.7-24.9)	1761	65.7↓		(63.8-67.4)
$40,001 to $60,000	126	5.5↓	(4.7-6.6)	284	12.5↓		(11.2-13.9)	1866	82.0↑		(80.4-83.5)
$60,000 to $80,000	96	4.2↓	(3.4-5.1)	232	10.1↓		(8.9-11.4)	1969	85.7↑		(84.3-87.1)
$80,001 or more	155	3.3↓	(2.8-3.9)	378	8.1↓		(7.4-8.9)	4127	88.6↑		(87.6-89.4)
Not stated	165	6.3	(5.4-7.3)	323	12.3↓		(11.1-13.6)	2140	81.4↑		(79.9-82.9)

SEIFA*											
Lowest quintile	191	7.5↑	(6.5-8.6)	429	16.8↑		(15.4-18.3)	1928	75.7↓		(74.0-77.3)
Low quintile	241	7.4↑	(6.5-8.3)	511	15.7		(14.5-17.0)	2504	76.9↓		(75.4-78.3)
Middle quintile	226	6.6	(5.9-7.5)	574	16.9↑		(15.7-18.2)	2598	76.5↓		(75.0-77.9)
High quintile	221	6.5	(5.7-7.3)	464	13.5↓		(12.4-14.7)	2741	80.0↑		(78.6-81.3)
Highest quintile	187	4.9↓	(4.3-5.7)	501	13.2↓		(12.2-14.3)	3103	81.9↑		(80.6-83.1)

**Overall**	1068	6.5	(6.1-6.9)	2482	15.1		(14.5-15.6)	12915	78.4		(77.8-79.1)

Note: The weighting of the data can result in rounding discrepancies or totals not adding

*Not stated category not reported

↓↑ Statistically significantly higher or lower (χ^2 ^test, p < 0.05) compared to other categories combined

Univariate odds ratios were then determined which compared the demographic characteristics of those who did not know the type of arthritis compared to those that did (Table 2). Those who did not know the type of arthritis that they had were more likely to be male, have a trade, certificate or diploma or secondary level of education, earn $20,001 to $40,000 or not state their income or be in the low or high SEIFA IRSD quintiles. Multivariate analysis indicated that males, those with have a trade, certificate or diploma or secondary level of education, who spoke a language other than English at home, were widowed and earned $20,001 to $60,000, more than $80,000 or did not state their income were more likely to state that they did not know what type of arthritis they had (Table 3).

**Table 2:** Univariate analysis of demographic variables for adults with arthritis but don't know type, South Australia, 2006-8

	n	%	OR	(95% OR)	p value
**Sex**					
Female	566/2140	26.5	1.00		
Male	501/1410	35.6	1.53	(1.33-1.77)	**< 0.001**
**Age**					
80 years and over	144/453	31.8	1.00		
60 to 79 years	463/1584	29.2	0.89	(0.71-1.11)	0.286
40 to 59 years	371/1242	29.9	0.91	(0.72-1.15)	0.441
18 to 39 years	90/271	33.2	1.06	(0.77-1.47)	0.704
Country of birth*					
Australia	783/2611	30.0	1.00		
UK/Ireland	138/505	27.4	0.88	(0.71-1.09)	0.239
Other	142/426	33.3	1.17	(0.94-1.45)	0.166
Language spoken at home*					
English	933/3160	29.5	1.00		
Other	130/382	34.0	1.23	(0.98-1.54)	0.069
Marital status*					
Married/live with partner	728/2459	29.6	1.00		
Separated/Divorced	93/336	27.5	0.90	(0.70-1.17)	0.439
Widowed	172/541	31.8	1.11	(0.91-1.35)	0.318
Never married	72/210	34.1	1.23	(0.92-1.66)	0.168
**Area of residence**					
Metropolitan	758/2532	29.9	1.00		
Country	310/1018	30.4	1.02	(0.87-1.20)	0.769
Education*					
Degree or higher	108/488	22.1	1.00		
Trade/Certificate/Diploma	267/832	32.0	1.66	(1.28-2.15)	**< 0.001**
No schooling up to secondary	690/2224	31.0	1.59	(1.26-2.00)	**< 0.001**
Employment*					
Economically inactive	644/2188	29.4	1.00		
Unemployed	19/53	36.3	1.36	(0.77-2.40)	0.282
Part time	148/495	29.9	1.02	(0.83-1.27)	0.828
Full time employed	256/813	31.5	1.10	(0.93-1.31)	0.274
**Household income**					
Up to $20,000	230/871	26.4	1.00		
$20,001-$40,000	296/921	32.2	1.32	(1.08-1.62)	**0.007**
$40,001-$60,000	126/410	30.7	1.24	(0.96-1.60)	0.107
$60,001-$80,000	96/327	29.2	1.15	(0.87-1.53)	0.324
More than $80,000	155/533	29.1	1.14	(0.90-1.45)	0.277
Not stated	165/488	33.8	1.42	(1.12-1.81)	**0.004**
SEIFA*					
Highest quintile	187/688	27.2	1.00		
High quintile	221/685	32.3	1.28	(1.01-1.61)	**0.039**
Middle quintile	226/800	28.2	1.05	(0.84-1.32)	0.661
Low quintile	241/751	32.0	1.26	(1.00-1.58)	**0.046**
Lowest quintile	191/620	30.8	1.74	(0.94-1.51)	0.153

*Not stated category not reported

**Table 3:** Multivariate analysis of demographic variables for adults with arthritis but don't know type, South Australia, 2006-8

	OR	(95% OR)	p value
**Sex**			
Female	1.00		
Male	1.65	(1.42-1.93)	**< 0.001**
Language spoken at home*			
English	1.00		
Other	1.28	(1.02-1.61)	**0.034**
Marital status*			
Married/live with partner	1.00		
Separated/Divorced	1.10	(0.84-1.44)	0.472
Widowed	1.49	(1.18-1.89)	**0.001**
Never married	1.32	(0.98-1.80)	0.071
Education*			
Degree or higher	1.00		
Trade/Certificate/Diploma	1.64	(1.26-2.14)	**< 0.001**
No schooling up to secondary	1.78	(1.39-2.28)	**< 0.001**
**Household income**			
up to $20,000	1.00		
$20,001-$40,000	1.50	(1.20-1.88)	**< 0.001**
$40,001-$60,000	1.46	(1.11-1.94)	**0.008**
$60,001-$80,000	1.36	(1.00-1.85)	0.051
More than $80,000	1.47	(1.11-1.94)	**0.007**
Not stated	1.60	(1.24-2.06)	**< 0.001**

*Not stated category not reported

Hosmer and Lemeshow goodness of fit Chi-square 4.45, p = 0.815

## Discussion

Population ageing continues and with it, an expected increase in arthritis prevalence in the future. This will result in a substantial increase in the number of Australians with arthritis over the next decade. While the self-reported prevalence of arthritis using telephone and questionnaire has been shown to have high agreement, the agreement is not as high when compared to clinical assessment [11,25]. These data have shown that there are a substantial number of adults with arthritis in the community who for a variety of reasons, do not understand the condition that they have. Previous data have also suggested that not only do respondents not know what type of arthritis they have, there is also poor validation of self-report for specific forms of arthritis, for example people report that they have RA, however when matched with medication records this is not the case [25]. This has ramifications for prevention messages, management and treatment regimes.

It is important to be able to accurately define the types of arthritis in the community, as the health service requirements of different subtypes are quite different, thus providing policy and program implications. For example, inflammatory arthritis (such as RA, ankylosing spondylitis, psoriatic arthritis, juvenile idiopathic arthritis/JRA) requires early referral to speciality rheumatology clinics and early intervention with disease modifying agents to minimise the burden of pain and disability. In contrast, treatment of degenerative arthritis (such as OA) requires multidisciplinary interventions including weight loss where appropriate, physiotherapy, education (including self-management courses) and, only in end-stage disease, orthopaedic surgical referral for joint replacement surgery [15]. By addressing causes and risk factors for arthritis, promoting healthier lifestyle choices and raising disease awareness in the general population [15] those with lower FHL may be better able to reduce arthritis risk and access appropriate interventions.

The prevalence of arthritis has remained relatively stable in SA and if all factors (for example, average age and life expectancy) are maintained at current levels, it is expected that the prevalence will remain unchanged (Figure 1). However, if the population continues to age and live longer, prevalence is then likely to rise, with similar trends being reported globally. For example, using 2003-2005 data, in the United States, it was estimated that 21.6% (or 46.4 million people) of the population aged 18 years and over had arthritis (which compares with the overall prevalence of arthritis of 21.6% obtained in this study) and this was estimated to increase to approximately 67 million people (an increase of 40%) by the year 2030 [14]. In England and Wales, between 1.3 and 1.75 million people are affected by OA and between 0.25 and 0.5 million people have RA [13]. The prevalence of arthritis in the Netherlands is 17.6% of the population aged 25 years or older [10]. In terms of Australian results, the self-reported prevalence of RA, OA and arthritis overall in SA are higher than national figures, however the ageing population profile in SA may account in part for this and also, particularly with regard to RA, the figure may be an overestimate due to confusion between rheumatism and RA [15].

This study shows that 30.1% of respondents with doctor diagnosed arthritis did not know what type of arthritis they had. This could be as a result of a variety of reasons including differences in definitions of arthritis. The description and meaning of different musculoskeletal diseases will differ between medical specialists, between medical specialists and the general public, and also between cultures and languages [9]. The condition of OA, in particular, raises some problems as there is no standard definition of OA used in all studies [14]. This is because OA can be clinically, radiographically or symptomatically defined and the prevalence is highly dependent on the definition used [9]. No national arthritis trend information exists that has used the same data source over time, emphasising the unique value of these South Australian surveillance data obtained using a consistent methodology.

There has been little change in the proportion of respondents reporting that they do not know the type of arthritis that they have over time. This may indicate that there have been few education programs related to arthritis or that they have had little impact. Thus there is likely to be little knowledge generally about the condition. Multivariate analysis indicated that variables associated with income, education, cultural diversity and sex were associated with not knowing the type of arthritis. We have not measured health literacy in this study. However, measures used in research are largely to measure the capacity and skills of people in being able to acquire and process information. The question of how well health literacy correlates with disease knowledge is unknown in this case but there is ample evidence elsewhere to indicate lower health literacy is associated with less disease knowledge [2,4,5,7]. Clinicians are unable to correctly identify those with limited FHL [26], and using educational level will misclassify a substantial proportion of people as health literate/illiterate [2,3,6,7,27-29]. Our data would strongly suggest explicitly taking into account the level of health literacy of individuals when communicating about arthritis may be critical in establishing understanding, particularly considering the multiple meanings different clinicians and others imply when they use the term "arthritis".

There are limitations to these data that may have had an effect on the observed prevalence of arthritis. First, the methodology of SAMSS requires that participants have a landline telephone number listed in the EWP. South Australians without a landline telephone, or without a landline number listed in the EWP are excluded in the sampling, which may result in an underestimate of arthritis prevalence given that arthritis is associated with increasing age. As many older people who could possibly have arthritis live in institutions without the availability of a personal landline, these estimates are likely to be an underestimation of the true problem. Secondly, although a representative sample of Aboriginal people are obtained in each SAMSS, the small absolute number of Aboriginal people surveyed prevent comparisons of arthritis prevalence trends between indigenous and non-indigenous groups. Third, the study includes only the self-reported prevalence of arthritis, that is, those people with doctor diagnosed arthritis. It has been documented that a higher prevalence is found for musculoskeletal diseases from self-reporting than when estimated from physical examinations [10,14,30] This has raised concerns over the validity of the self-reporting of these conditions and it has been argued that the validity of the self-reporting of musculoskeletal conditions is poor when the figures are compared with physical examination [10]. In addition, Lawrence et al [31] presents concerns over the severe limitations with regard to estimating prevalence of specific conditions (using self-report data) due to the fact that individuals frequently do not know and therefore cannot identify the specific musculoskeletal disease that affects them. However, it is concluded that self-report data are better used to identify the more generic condition of "arthritis". In order to overcome this problem of definition in establishing the prevalence of arthritis in the population, the consensus of a working group of experts was that "symptomatic arthritis" rather than "radiographic evidence of arthritis" should be used to measure prevalence [31]. Symptomatic includes both self-reported arthritis as well as reported pain in the joints [10]. The data presented in this paper are solely based on self-report and the limitations associated with this data collection (as discussed above) are acknowledged.

## Conclusions

Continued surveillance, using population tools such as the South Australian Monitoring and Surveillance System, will monitor key arthritis indicators. The ultimate aim of collection and analysis of surveillance data is to detect population changes at an early stage, to inform policy and program decisions that initiate action. While this paper focuses on a single condition, health literacy is an important aspect in implementing actions across the spectrum of health care issues. The link from surveillance to public health practice is essential [15], not only if the health outcomes of the population who have arthritis are to be improved, but also if the general population is to be prevented from developing arthritis and the upward trend in arthritis prevalence is to be curtailed.

## Competing interests

The authors declare that they have no competing interests.

## Authors' contributions

Each author has contributed intellectually to this work

TKG statistical analysis, interpretation of data, writing manuscript; CLH, RJA, DB, JB AWT interpretation of data, writing manuscript. All authors read and approved the final manuscript.

## Pre-publication history

The pre-publication history for this paper can be accessed here:

http://www.biomedcentral.com/1471-2474/11/174/prepub
